# Genito-Urinary Surgery

**Published:** 1895-12-14

**Authors:** 


### GENITO-URINARY SURGERY.

**Strangulation of the Testes.**—Dr. John V. D. Poel (New York) has collected[1] all the recorded cases of strangulation of the testes from torsion of the cord. Torsion in the majority of cases occurs when the testis lies in the inguinal canal or has not reached the bottom of the scrotum; and the normal fixation of the organ by the peritoneal reflex must be absent. In some of the cases the symptoms came on when the patients were in bed, in others after some violent exertion. The testis and epididymis swells and becomes of a bluish-black colour. In some cases it sloughs, and in others it only atrophies when the cord has been untwisted by operation. The degree of torsion sufficient to cause strangulation has varied from a half turn to two full turns. Many times the condition, often associated as it is with incomplete descent of the testis, has been mistaken for strangulated hernia and operated on for such. The black testis, with its twisted cord looking like leeches, has then been found, and supposed at first to be gangrenous bowel. There is pain, local swelling, perhaps œdema, redness and fever, and sometimes vomiting; but the symptoms of complete obstruction present in strangulated hernia not absent. If the condition can be diagnosed early, the cord might be untwisted in time to save the testis. Whether the testis be left or removed when operated on later in the case will depend on the degree of strangulation.

Experimental ligature of the various constituents of the cord has given varying results in the hands of different workers. Alfessandri[2] finds that after ligature of the vas deferens the testis and epididymis eventually atrophy, and the same result follows ligature of the spermative artery and the pampiniform plexus, and if the artery of the vas is included the atrophy is more rapid. Ligature of the artery of the vas alone has no effect on the testis or epididymis. After excision of the nerves there was no naked eye change, but a coagulative necrosis set in.

**Syphilis.**—Various attempts have lately been made to cure syphilis by the use of serum of animals inoculated, or of animals which are refractory to syphilitic infection.[3] Fournier and Feulard, of the Hôpital St. Louis, have been trying the latter method, but without success; Richet and Héricourt the former, with some benefit in the few cases in which they have tried it. Others, again, have used the serum of syphilitic patients in the secondary stage, on the theory that serum from such sources may be expected to contain the products of syphilitic secretion, and have an anti-toxic property analogous to diphtheria anti-toxine.

The infectious nature of congenital syphilis is discussed by R. W. Parker and others.[4] Mr. Parker, who has had a very large experience of the disease, says he can scarcely remember to have seen a single reliable case of infection by a congenitally syphilitic infant, though he has seen numerous instances of suckling of such infants by healthy women other than their mothers.

[1] Medidal Record, June 15, 1895, p. 737. [2] Il Policlinicfo, May 1, 1895. Epitome B.M.J., June 8, 1895. [3] Progrès Méd., No. 15, 1895, Quarterly Review, July, 1895, Richet Soc. de Biol., Jan. 12, 1895, Epit. B.M.J. March 23, 1895, Gilbert and Furnier, Sem. Méd., April 27, 1895, and Epi, B.M.J., June 1, 1895. [4] Brit. Med. J., Feb. 9, 1895.

